# Common Risk Factors Associated With Chronic Kidney Disease in Sudan: A Pilot Study

**DOI:** 10.7759/cureus.98780

**Published:** 2025-12-09

**Authors:** Eldisugi Hassan M Humida, Awad Mohamed, Suliman A Ahmed, Amal Khalil Y Mohammed, Namarig Alhadi Hamid, Hussain G Ahmed

**Affiliations:** 1 Medicine, University of Kordofan, El-Obeid, SDN; 2 Cardiac Catheterisation Laboratory, El-Obeid International Hospital, El-Obeid, SDN; 3 Cardiology, St. Luke's General Hospital, Kilkenny, IRL; 4 Medicine, University of Khartoum, Khartoum, SDN; 5 Medicine, El-Obeid International Hospital, El-Obeid, SDN; 6 Medicine, El-Obeid Teaching Hospital, El-Obeid, SDN; 7 Pathology, Prof Medical Research Consultancy Center, El-Obeid, SDN; 8 Histopathology and Cytology, University of Khartoum, Khartoum, SDN

**Keywords:** chronic kidney disease, diabetes, hypertension, obesity, sudan

## Abstract

Background: Kidney failure is common in Sudan, usually preceded by chronic kidney disease (CKD). The purpose of this study was to identify the most common risk factors associated with CKD in Sudan.

Methodology: This was a cross-sectional descriptive clinic-based study undertaken in El-Obeid, the capital of North Kordofan state. Because of the ongoing armed war and the failure of the healthcare system in most places in the state, practically everyone in the state seeks medical care in El-Obeid. Selection of applicants is based on the individual's willingness to participate in the study.

Results: Hypertension was detected in 8.2% of the subjects, among whom 14.3% exhibited a glomerular filtration rate (GFR) of <60 mL/min/1.73 m². Cigarette smoking revealed findings analogous to hypertension. Among the 15.8% of diabetic individuals, 22.2% showed a GFR of <60 mL/min/1.73 m². Urinary tract infection was diagnosed in 11% of patients, with 21% exhibiting a GFR of <60 mL/min/1.73 m².

Conclusion: Risk factors for CKD, including diabetes, hypertension, tobacco use, urinary tract infections, and obesity, are common in western Sudan. These risks require proactive intervention from both the healthcare system and the community.

## Introduction

The International Society of Nephrology (ISN) Global Kidney Health Atlas (GKHA) has made significant advancements in establishing a multinational initiative aimed at presenting a clear overview of kidney care delivery in low-income and other countries. This effort focuses on ensuring access to essential medicines, kidney replacement therapy (KRT), and conservative kidney management [1].

Chronic kidney disease (CKD) ranks among the most severe non-communicable diseases globally, with incident cases rising from 7.80 million in 1990 to 18.90 million in 2019. Concurrently, the disability-adjusted life years (DALYs) associated with CKD increased from 21.50 million to 41.54 million. Although global mortality from CKD is anticipated to decline, the incidence of new cases is projected to increase by 2030 [2,3]. The Kidney Disease Improving Global Outcome (KDIGO) guidelines classify kidney disease based on glomerular filtration rate (GFR) and albuminuria to estimate outcomes [4].

Globally, several risk factors have been associated with the initiation and progression of CKD, including type 2 diabetes mellitus (T2DM), hypertension [5,6], tobacco use [7], obesity [8], and urinary tract infection (UTI) [9]. Diabetic kidney disease markedly elevates the risk of cardiovascular events (CVEs), with the majority of fatalities attributed to cardiovascular complications [5,6].

Oxidative stress and inflammation of the nephrons and renal tubules are significant contributors to hypertension, resulting in conditions such as nephrosclerosis and hypertensive nephropathy. The environment and genetic factors significantly contribute to hypertensive kidney damage, with African Americans being particularly susceptible [10].

The armed conflict in Sudan, which began on April 15, 2023, has significantly worsened the suffering of the population due to the near collapse of the health system, particularly affecting the availability of essential health services such as dialysis and other daily kidney care [11]. Numerous individuals who appear healthy may actually have progressing kidney disease, and many of these individuals are potential candidates for end-stage renal disease (ESRD). However, the literature on CKD in Sudan is limited.

The objectives of this study were to measure proportions of the risk factors for CKD including diabetes, hypertension, tobacco use, UTI, and obesity in western Sudan.

## Materials and methods

**Figure 1:**
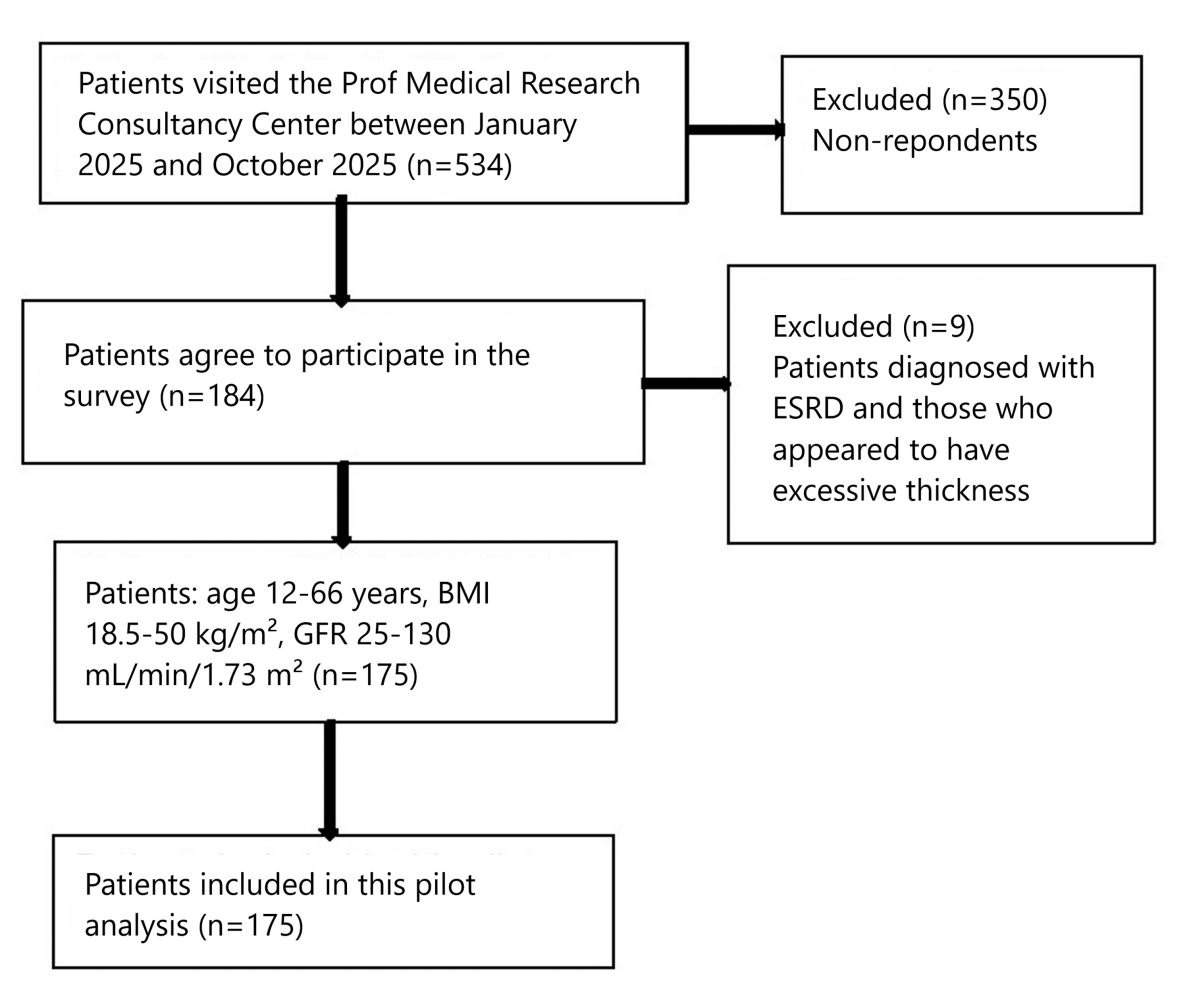
Cohort flowchart illustrating the patient selection process ESRD: end-stage renal disease; GFR: glomerular filtration rate

This study was a cross-sectional descriptive clinic-based study carried out in El-Obeid city, the capital of North Kordofan state. The ongoing armed conflict has led to a situation where nearly all individuals from other cities within the state are seeking healthcare services in El-Obeid. The research was carried out at Prof Medical Research Consultancy Center, El-Obeid, Sudan, from January 2025 to October 2025 after obtaining approval from the institute's Human Research Ethics Committee (HREC) (approval number: HREC 0020/MRCC.7/25). Participants were chosen according to their willingness to participate in the study. All individuals who visited our healthcare clinics for a general checkup and agreed to participate were randomly selected, irrespective of any demographic characteristics. Individuals diagnosed with ESRD and those who appeared to have excessive thickness were excluded from the study (see Figure 1). Individuals with a lack of urinalysis, at least one tertiary nephrology consultation, dialysis or transplantation, and acute renal failure were excluded [12]. Diabetes [13], hypertension [14], obesity [15], and UTI [16] were diagnosed based on corresponding studies. The CKD stages were considered according to National Kidney Foundation guidelines [17], as follows: stage 1: eGFR ≥90 mL/min/1.73 m²; stage 2: eGFR 60-89 mL/min/1.73 m²; stage 3a: eGFR 45-59 mL/min/1.73 m²; stage 3b: eGFR 30-44 mL/min/1.73 m²; stage 4: eGFR 15-29 mL/min/1.73 m²; and stage 5: eGFR <15 mL/min/1.73 m². As this investigation is a pilot, we are planning to examine a sample size of approximately 2000 individuals.

Statistical analysis

The collected data were first organized in a data sheet and subsequently entered into IBM SPSS Statistics for Windows, Version 31.0 (IBM Corp., Armonk, New York, United States). Percentages, frequencies, and tabulations were computed. Relative risk (RR) and chi-squared tests were computed. A p-value below 0.05 was deemed statistically significant.

## Results

**Figure 2:**
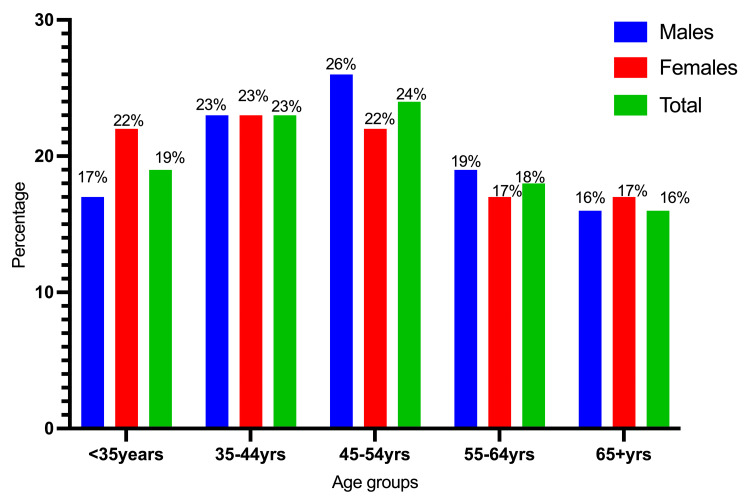
Description of the participants by gender and age

**Figure 3:**
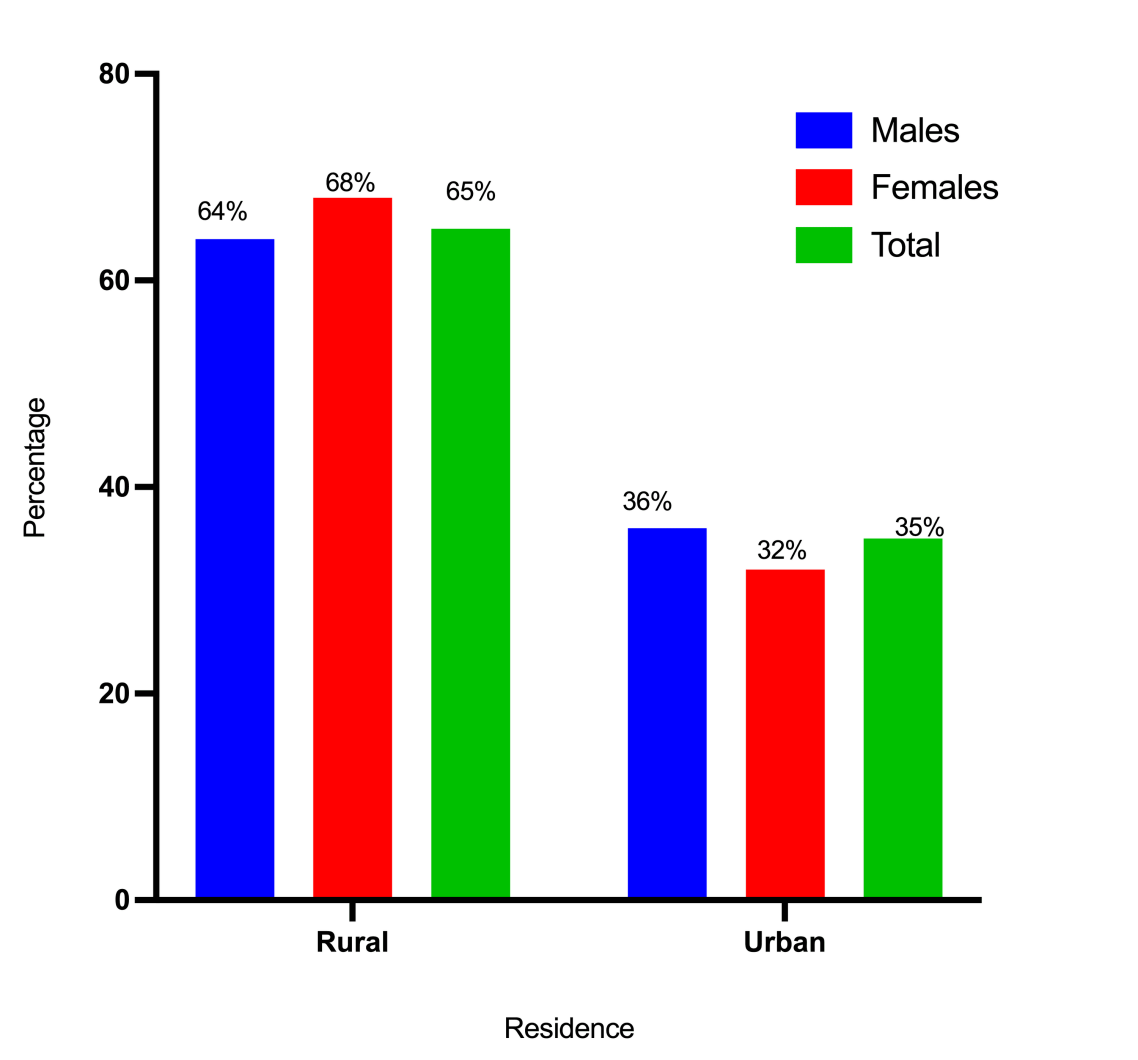
Description of the participants by gender and residence

**Figure 4:**
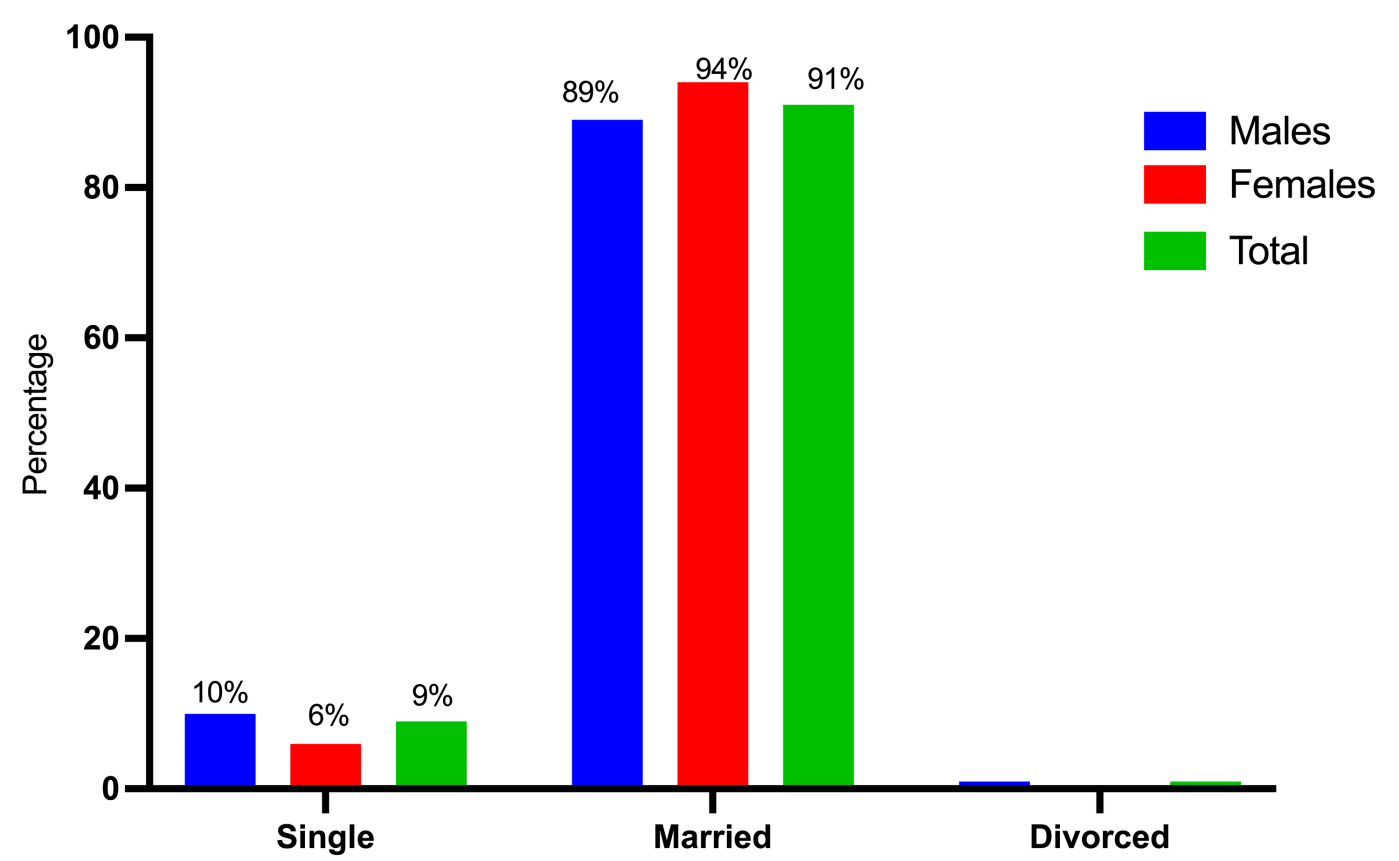
Description of the participants by gender and marital status

A total of 175 participants were involved in this study, ranging in age from 12 to 102 years, with a mean age of 48±16 years. Among the 175 participants, 110 (62.9%) were men, and 65 (37.1%) were women. The majority of the study population fell within the age range of 45-54, followed by those aged 35-44 and those under 35 years, with incidence rates of 42 (24%), 40 (23%), and 33 (19%), respectively. A majority of the participants, 114 out of 175 (65%), were from rural areas, whereas the remaining 61 (35%) were from urban areas. A significant portion of the study subjects, specifically 159 out of 175 (90.8%), were married (Table 1 and Figures 2-4).

**Table 1:** Participant distribution categorized by sex, age, residence, and marital status

Category	Variable	Males (n=110)	Females (n=65)	Total (n=175)
Age (in years)	<35 years	19 (17.3%)	14 (21.5%)	33 (19%)
	35-44	25 (22.7%)	15 (23%)	40 (23%)
	45-54	28 (25.5%)	14 (21.5%)	42 (24%)
	55-64	21 (19%)	11 (17%)	32 (18%)
	65+	17 (15.5%)	11 (17%)	28 (16%)
Residence	Rural	70 (63.6%)	44 (67.7%)	114 (65%)
	Urban	40 (36.4%)	21 (32.3%)	61 (35%)
Marital status	Single	11 (10%)	4 (6.2%)	15 (8.6%)
	Married	98 (89%)	61 (93.8%)	159 (90.8%)
	Divorced	1 (1%)	0 (0%)	1 (0.6%)

**Figure 5:**
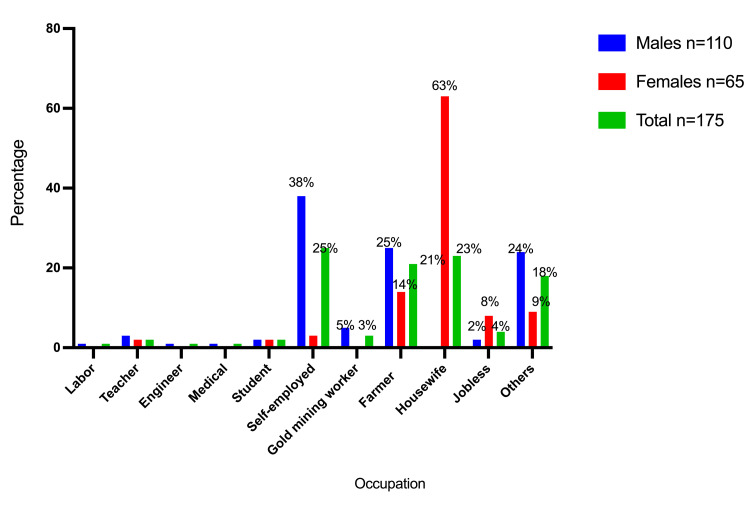
Description of the participants by gender and occupation

**Figure 6:**
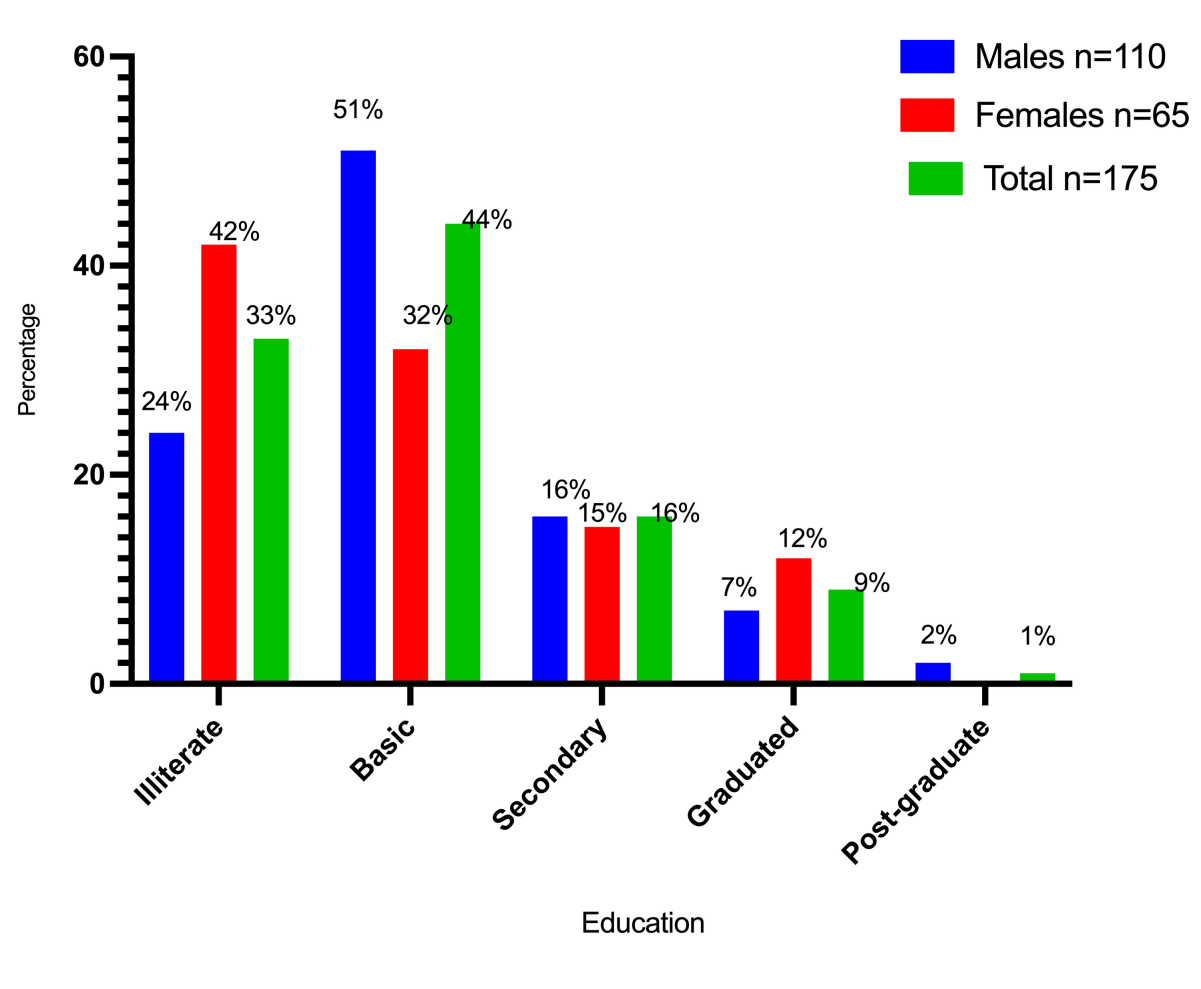
Description of the participants by gender and education

**Figure 7:**
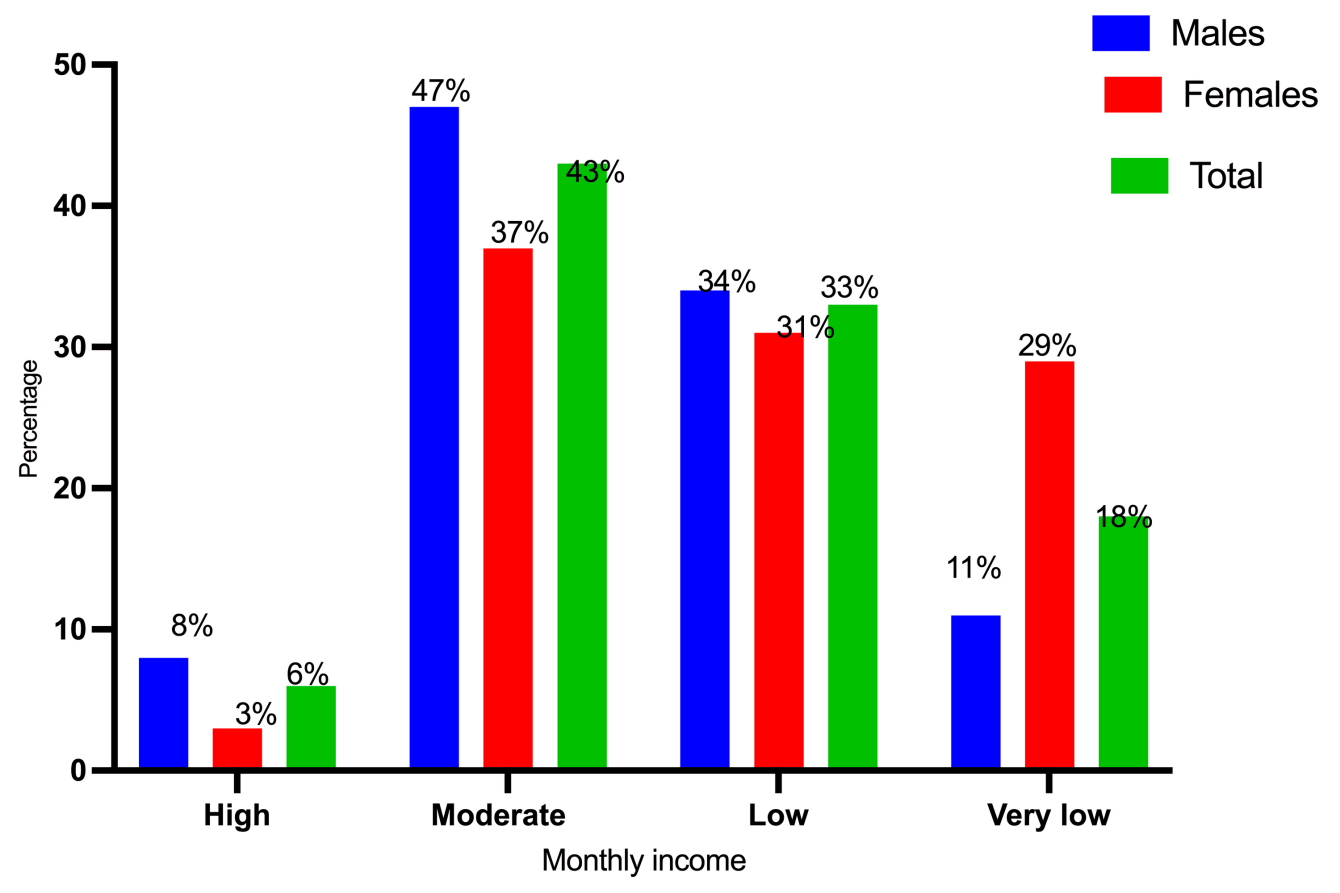
Description of the participants by gender and financial status

Table 2 and Figures 5-7 summarize the participant distribution by gender, occupation, education, and financial level. Most of them were self-employed, followed by housewives and farmers, who accounted for 44 (25%), 41 (23.3%), and 36 (20.6%), respectively. The bulk of the participants had a basic level of education (77 (44%)), followed by illiterates (52 (29.7%)) and those with a secondary level of education (28 (16%)). Most of them had a moderate financial position, followed by low financial status and destitute financial status, which accounted for 76 (43.4%), 57 (32.6%), and 31 (17.7%), respectively.

**Table 2:** Participant distribution categorized by sex, employment, education, and financial condition

Category	Variable	Males (n=110)	Females (n=65)	Total (n=175)
Occupation	Labor	1 (0.9%)	0 (0%)	1 (0.6%)
	Teacher	3 (2.7%)	1 (1.5%)	4 (2.3%)
	Engineer	1 (0.9%)	0 (0%)	1 (0.6%)
	Medical	1 (0.9%)	0 (0%)	1 (0.6%)
	Student	2 (1.8%)	1 (1.5%)	3 (1.7%)
	Self-employed	42 (38.2%)	2 (3.1%)	44 (25%)
	Gold mining worker	5 (4.5%)	0 (0%)	5 (2.9%)
	Farmer	27 (24.5%)	9 (13.8%)	36 (20.6%)
	Housewife	0 (0%)	41 (63%)	41 (23.3%)
	Jobless	2 (1.8%)	5 (7.7%)	7 (4%)
	Others	26 (23.6%)	6 (9.2%)	32 (18.3)
Education	Illiterate	26 (23.6%)	26 (41.9%)	52 (29.7%)
	Basic	56 (50.9%)	21 (32%)	77 (44%)
	Secondary	18 (16.4%)	10 (15.4%)	28 (16%)
	Graduated	8 (7.3%)	8 (12%)	16 (9.1%)
	Post-graduate	2 (1.8%)	0 (0%)	2 (1%)
Financial status	High	9 (8.2%)	2 (3.1%)	11 (6.3%)
	Moderate	52 (47.3%)	24 (36.9%)	76 (43.4%)
	Low	37 (33.6%)	20 (30.8%)	57 (32.6%)
	Very low	12 (10.9%)	19 (29%)	31 (17.7%)

The GFR calculation for this study population revealed that 1/175 (0.6%) patient was detected with stage 5, 1/175 (0.6%) with stage 4, 7/175 (4%) with stage 3a, 24/175 (13.7%) with stage 3b, 80/175 (45.7%) with stage 2, and 62/175 (35.4%) with stage 1.

**Figure 8:**
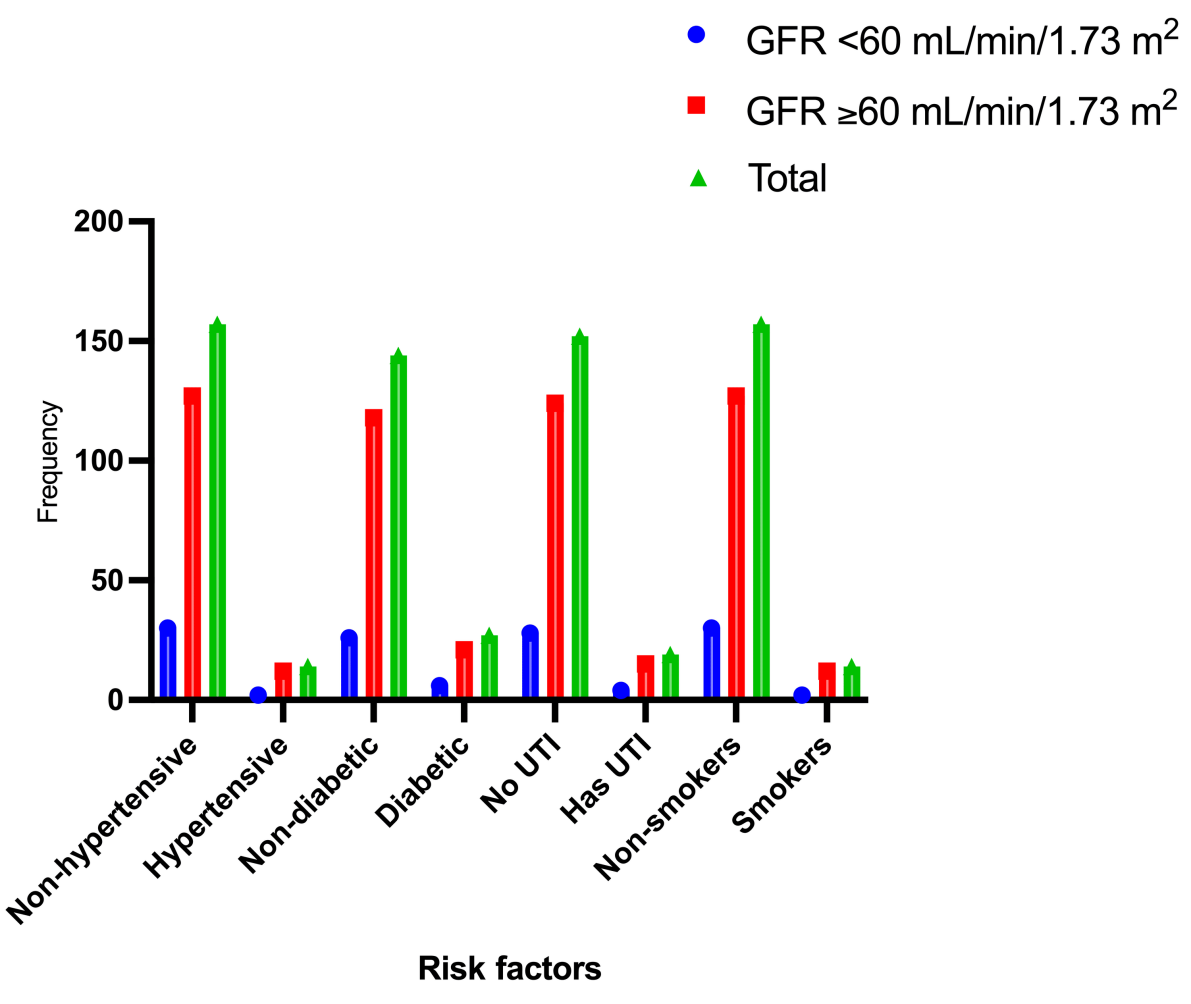
Description of the participants by CKD risk factors CKD: chronic kidney disease; GFR: glomerular filtration rate; UTI: urinary tract infection

Table 3 and Figure 8 describe the study subjects' distribution based on CKD risk factors. Of the 171 participants, 14 (8.2%) had hypertension, with 2/14 (14.3%) having a GFR of <60 mL/min/1.73 m². Cigarette smoking produced identical results as hypertension. Of the 27/171 (15.8%) diabetic individuals, 6/27 (22.2%) had GFR <60 mL/min/1.73 m². The risk of diabetes mellitus in developing CKD is as follows: adjusted odds ratio (OR)=1.2411; 95% CI: 0.5456-2.8232; and Pearson's chi-squared test p=0.6065. Of the 171 subjects, 19 (11%) had UTI, with 4/19 (21%) having a GFR <60 mL/min/1.73 m². The risk of UTI in conjunction with CKD is as follows: OR=1.2833; 95% CI: 0.4558-3.6132; and p=0.6367.

**Table 3:** Participant distribution according to CKD risk variables CKD: chronic kidney disease; GFR: glomerular filtration rate

Category	Variable	GFR <60 mL/min/1.73 m^2 ^(n=32)	GFR ≥60 mL/min/1.73 m^2^ (n=139)	Total (n=171)
Hypertension	No	30 (93.7%)	127 (91.4%)	157 (91.8%)
	Yes	2 (6.3%)	12 (8.6%)	14 (8.2%)
Diabetes	No	26 (81.3%)	118 (84.9%)	144 (84.2%)
	Yes	6 (18.8%)	21 (15%)	27 (15.8%)
Urinary tract infection	No	28 (87.5%)	124 (89.2%)	152 (88.9%)
	Yes	4 (12.5%)	15 (10.8%)	19 (11%)
Cigarette smoking	No	30 (93.8%)	127 (91.4%)	157 (91.8%)
	Yes	2 (6.3%)	12 (8.3%)	14 (8.2%)

**Figure 9:**
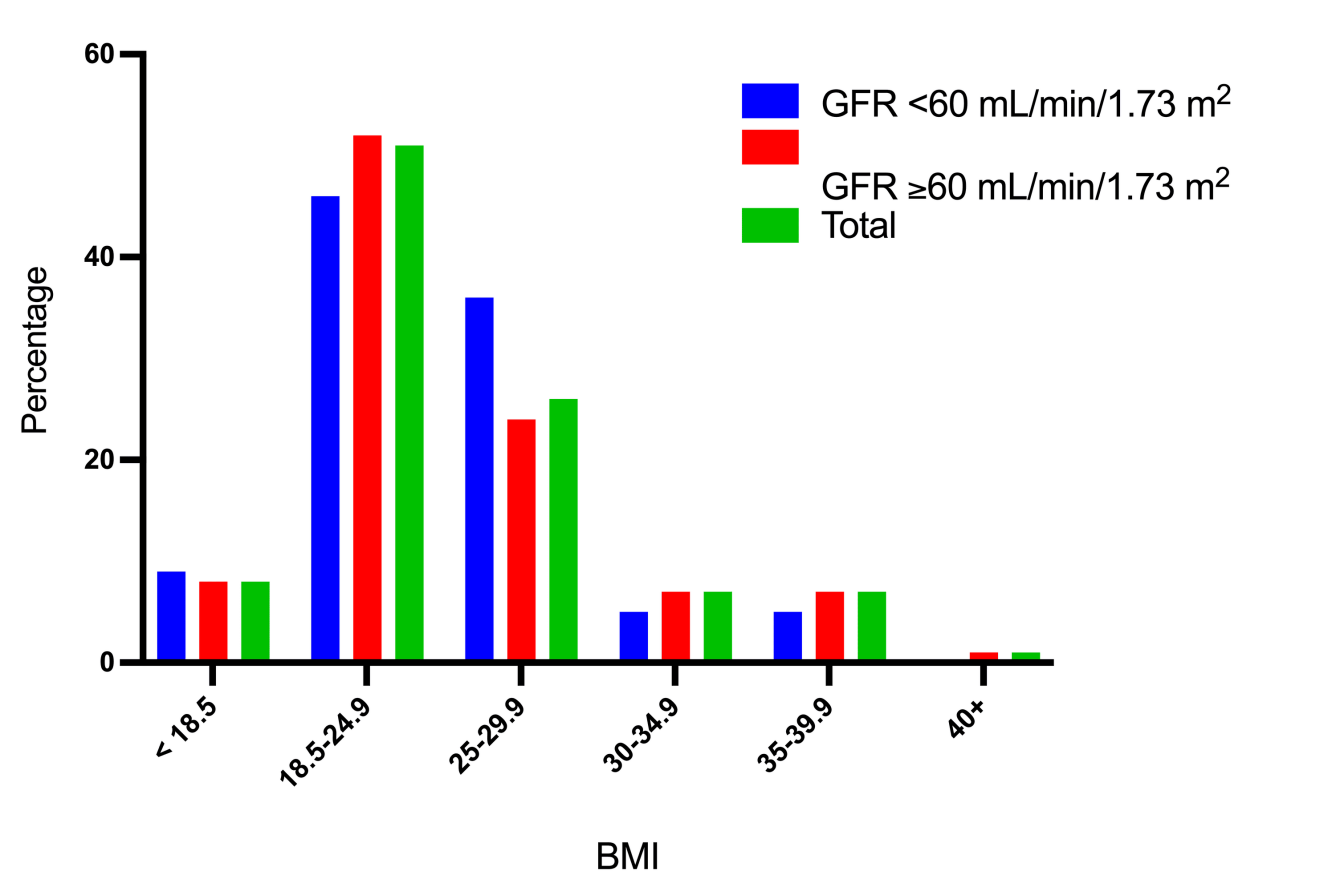
Description of the participants by GFR level and BMI GFR: glomerular filtration rate

**Figure 10:**
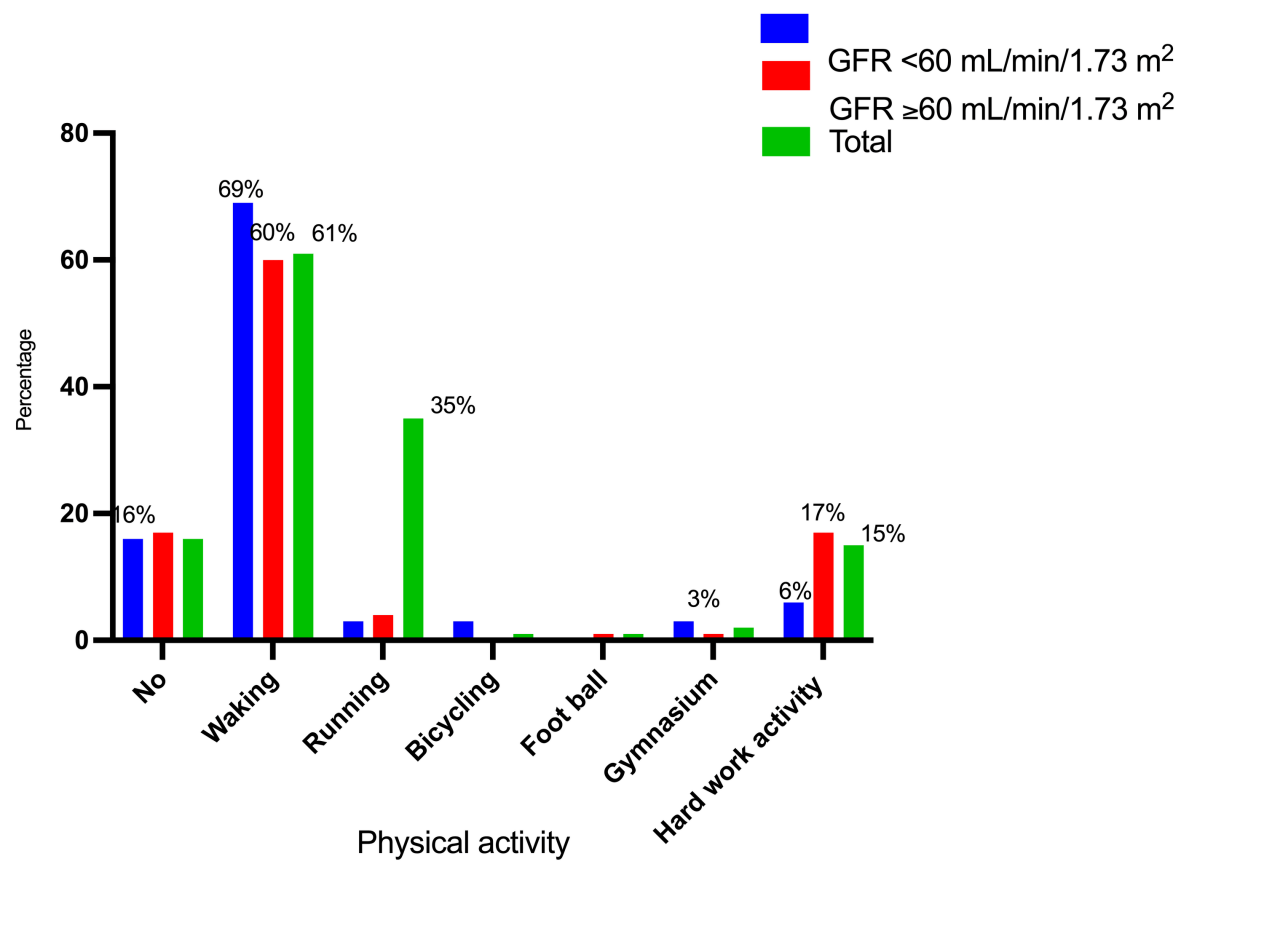
Description of the participants by GFR level and physical activity status GFR: glomerular filtration rate

Table 4 and Figures 9-10 describe the study subjects' distribution by BMI and physical activity status. Of the 22/143 (15.4%) persons with GFR <60 mL/min/1.73 m², 10 (13.7%) were among those with a BMI of 18.5-24.9, and 8/37 (21.6%) were among those with a BMI of 25-29.9. Around 32/171 (18.7%) individuals with GFR <60 mL/min/1.73 m² reported physical activity, with 22/27 (81.5%) indicating walking and 2/27 (7.4%) indicating hard work activity.

**Table 4:** Participant distribution categorized by BMI and physical activity status GFR: glomerular filtration rate

Category	Variable	GFR <60 mL/min/1.73 m^2^	GFR ≥60 mL/min/1.73 m^2^	Total
BMI	<18.5	2 (9.1%)	10 (8.3%)	12 (8.4%)
	18.5-24.9	10 (45.5%)	63 (52%)	73 (51%)
	25-29.9	8 (36.4%)	29 (24%)	37 (25.9%)
	30-34.9	1 (4.5%)	9 (7.4%)	10 (7%)
	35-39.9	1 (4.5%)	9 (7.4%)	10 (7%)
	40+	0 (0%)	1 (0.8%)	1 (0.7%)
	Total	22 (100%)	121 (100)	143 (100%)
Physical activity	No	5 (15.6%)	23 (16.5%)	28 (16.4%)
	Waking	22 (68.8%)	83 (59.7%)	105 (61.4%)
	Running	1 (3.1%)	5 (3.6%)	6 (35%)
	Bicycling	1 (3.1%)	0 (0%)	1 (0.6)
	Foot ball	0 (0%)	2 (1.4%)	2 (1.2%)
	Gymnasium	1 (3.1%)	2 (1.4%)	3 (1.8%)
	Hard work activity	2 (6.3%)	24 (17.3%)	26 (15.2%)
	Total	32 (100%)	139 (100%)	171 (100%)

## Discussion

CKD is a significant global health concern, impacting numerous individuals of reproductive age, and it carries substantial social and psychological implications for families and communities. The ongoing conflict in Sudan has significantly worsened the already fragile health system, leading to a critical shortage of health services, especially for patients suffering from CKD and ESRD.

The results of this study indicated substantial proportions of advanced stages of CKD. Although substantial data exist concerning the epidemiology of CKD in various African nations, information on this topic remains limited for Sudan, the third-largest country on the continent. A recent study conducted in Sudan indicated that approximately 9.4% of adults in central Sudan are affected by CKD. Researchers correlated CKD with older age and the use of diuretics [18].

CKD patients are vulnerable, as shown by recent violent confrontations. In war-torn Ethiopia, Sudan, Gaza, and Ukraine, basic services are unavailable. Antihypertensive and antidiabetic drugs are commonly unavailable to CKD patients. Damage to centers or supply chains increases dialysis patient mortality. Over 65% of Sudanese hemodialysis patients had problems from missing sessions. Wartime sources in Bosnia, Ethiopia, and Gaza claimed renal failure death rates around or above 50%. However, Ukraine has proved that dialysis services can be maintained and increased with intact infrastructure and international backing. These experiences demonstrate the importance of logistics, preparedness, and humanitarian coordination in renal care during conflict [19].

In 1990, 378 million (354-407) individuals had CKD; in 2023, 788 million (95% uncertainty interval 743-843) did. Adult CKD prevalence worldwide was 14.2% (13.4-15.2), a 3.5% (2.7-4.1) increase from 1990. The region with the highest age-standardized frequency was North Africa and the Middle East (18%; 16.9-19.4). Most individuals had stage 1-3 CKD, with a total frequency of 13.9% (13.1-15%). Globally, CKD was the ninth major cause of death in 2023, causing 1.48 million deaths (1.30-1.65), and the 12th largest cause of DALYs (769.2 (691.8) to 857.4 per 100,000). Poor renal function was a risk factor for 11.5% (8.4-14.5) of cardiovascular fatalities. Fasting plasma glucose, BMI, and systolic blood pressure were major CKD DALY risk variables [20].

Furthermore, there is a lack of literature concerning the relationship between CKD and various risk factors. The majority of reports from Sudan focused on ESRD or renal failure.

The current investigation found multiple CKD risk factors, with T2DM accounting for 15% of the patients. Diabetes is a significant risk factor for various complications, including renal and cardiovascular problems. The global and regional rise in diabetes, particularly among younger populations, correlates with an increased prevalence of kidney disease. T2DM-associated CKD prevention necessitated a deeper understanding of the pathophysiology of those at high risk for the disease, as well as ongoing lifestyle changes. Early aggressive combination therapy for diabetic kidney disease reduces the progression of CKD to ESRD, especially if it begins at an early stage, potentially reversing renal function decline [21-24].

In the current dataset, hypertension is recorded in 8% of cases. The World Health Organization found an increase in the prevalence of systemic arterial hypertension, which contributed considerably to increased cardiovascular mortality. Hypertension contributes significantly to the initiation and progression of CKD through a variety of mechanisms, including decreased renal perfusion, salt and water retention, renin-angiotensin-aldosterone system (RAAS) and sympathetic nervous system activation, and vascular endothelial damage, resulting in extracellular volume expansion and an increase in peripheral vascular resistance. Although long-term poorly controlled arterial hypertension can lead to deterioration in kidney function, CKD also has a negative impact on optimal hypertension control, resulting in a worsening of the kidney profile, necessitating continuous blood pressure monitoring, with home and ambulatory blood pressure measurement (ABPM) being the standard of care [25-27]. UTI was found in 11% of the cases in this study. Many studies have shown an increase in the risk of progression and deterioration in CKD stages, particularly in immunosuppressed people and transplant recipients, whereas UTI and pyelonephritis can cause renal scarring, which may be a trigger for future CKD development and progression [28].

In this study, 8% of individuals acknowledged smoking. Smoking is a substantial risk factor for death from various illnesses, including CKD. It exacerbates the effects of other risk factors such as diabetes, proteinuria, and GFR decline. Cotinine is a metabolite of nicotine, the primary psychoactive component of both tobacco and electronic cigarettes (e-cigarettes), that is then converted in the bloodstream to the active metabolite hydroxycotinine, which can remain in the bloodstream for up to 48 hours and aids in the self-verification of current smoking status. Smoking is a well-known risk factor for CKD, and individuals who smoke their first cigarette shortly after awakening have a greater chance of incident CKD [8,29].

We found that 26% of the cases in this group were overweight, with 15% being obese. Obesity remains a global health concern, and the number of obese people has increased in recent decades. It is a significant risk factor for several diseases, including CKD onset and development. The interrelationships between obesity and CKD are complex; however, chronic inflammatory process, oxidative stress, hemodynamic effect, and RAAS activation were the most common etiological factors, with glomerulomegaly, microalbuminuria, renal scarring, and glomerulosclerosis as the main pathological findings. Although weight loss and RAAS blockage may halt or reverse CKD, many people will eventually acquire ESRD. The connection of BMI and weight with mortality in CKD patients remains debatable; nevertheless, higher survival is established in ESRD [30,31].

In the current study, 83% of participants reported physical activity, with walking accounting for 61% of the total. Physical activity and exercise have been shown to reduce cardiovascular risk in CKD patients, potentially improving all cardiometabolic risk variables; however, the dose and style of exercise should be customized to the individual's health state and comorbidities. The type and intensity of exercise clearly correlate with cardiorespiratory fitness and body composition across the CKD spectrum. Some studies found better renal outcomes in both diabetic and non-diabetic CKD stage 3b patients, as well as better survival in non-diabetic stage 3b-5 CKD patients, supporting the concept of tailored physical activity based on disease stage and individual comorbid risk factors [32,33].

The physiological mechanisms behind the therapeutic effects of physical activity on different chronic diseases, including cardiovascular disease, diabetes mellitus, obesity, and mental health disorders, are generally recognized [34,35]. Engaging in regular physical activity yields numerous health advantages, enhancing sleep quality and alleviating symptoms associated with sleep disorders. Given the established advantages of moderate-intensity activities for sleep quality, there is an increasing interest in employing physical activity as an effective therapeutic strategy for various sleep disorders [36].

The current study carries multiple strengths, since the screening for CKD within the Sudanese community includes various advantages that may facilitate significant health improvements. The study can substantially impact public health initiatives and improve healthcare delivery for CKD in Sudan by emphasizing early detection, social engagement, and extensive utilization of data.

The current study, while providing initial data on the correlation between the rising prevalence of kidney failure and its associated risk factors, has certain limitations, notably its pilot status.

## Conclusions

The risk factors for CKD are widespread in western Sudan, with diabetes, hypertension, tobacco smoke, UTI, and obesity being the most frequent, requiring proactive intervention from both the healthcare system and the population.
